# NeuMa - the absolute Neuromarketing dataset en route to an holistic understanding of consumer behaviour

**DOI:** 10.1038/s41597-023-02392-9

**Published:** 2023-08-03

**Authors:** Kostas Georgiadis, Fotis P. Kalaganis, Kyriakos Riskos, Eleftheria Matta, Vangelis P. Oikonomou, Ioanna Yfantidou, Dimitris Chantziaras, Kyriakos Pantouvakis, Spiros Nikolopoulos, Nikos A. Laskaris, Ioannis Kompatsiaris

**Affiliations:** 1 Centre for Research & Technology Hellas, Information Technologies Institute (ITI), Thermi-Thessaloniki, Greece; 2 Aristotle University of Thessaloniki, Department of Journalism and Mass Communications, Thessaloniki, Greece; 3 Erasmus University Rotterdam, Department of Media and Communication, Rotterdam, the Netherlands; 4 D. Masoutis S.A., Thessaloniki, Greece; 5 MMS Advertising – Full Service Agency, Thessaloniki, Greece; 6 Artificial Intelligence & Information Analysis Lab, Department of Informatics, School of Sciences, Aristotle University of Thessaloniki, Thessaloniki, Greece

**Keywords:** Decision, Data processing

## Abstract

Neuromarketing is a continuously evolving field that utilises neuroimaging technologies to explore consumers’ behavioural responses to specific marketing-related stimulation, and furthermore introduces novel marketing tools that could complement the traditional ones like questionnaires. In this context, the present paper introduces a multimodal Neuromarketing dataset that encompasses the data from 42 individuals who participated in an advertising brochure-browsing scenario. In more detail, participants were exposed to a series of supermarket brochures (containing various products) and instructed to select the products they intended to buy. The data collected for each individual executing this protocol included: (i) encephalographic (EEG) recordings, (ii) eye tracking (ET) recordings, (iii) questionnaire responses (demographic, profiling and product related questions), and (iv) computer mouse data. NeuMa dataset has both dynamic and multimodal nature and, due to the narrow availability of open relevant datasets, provides new and unique opportunities for researchers in the field to attempt a more holistic approach to neuromarketing.

## Background & Summary

Neuromarketing^1^ refers to the emerging field that lies at the intersection of consumer behaviour studies and neuroscience. Using a more strict definition, neuromarketing refers to the “application of neuroscience in the marketing field” and plays an instrumental role in understanding how consumers’ decisions are driven by the subconscious mind. Despite the initial scepticism^2^, neuromarketing has met a rapid growth in the last years, as neuroimaging technology fosters the investigation of cognitive activity that may not be consciously perceived by the consumers^3^.

Recent innovations in long-standing science and neuroimaging technology have brought neuroscientists and marketers to a common ground that allows the integration of marketing with neuroscience. Within this context, a recent key study^4^ underlined the field’s two most significant issues. Firstly, the vast majority of studies are confined to studying consumers’ brains irrespectively from consumers’ behaviour, limiting their conclusions to correlational but not causal evidence. Secondly, typical neuromarketing studies are based on the assumption that a brain region is causally associated with a cognitive process. In other words, these studies often conclude based on reverse inference, e.g., that participants exhibit a particular psychological state based on the observed neural activation in a particular brain region.

Among the existing neuroimaging methods, electroencephalography (EEG) emerges as the least invasive and most affordable solution. Despite the fact that its spatial resolution is inferior compared to other neuroimaging technologies (e.g., fMRI, fNIRS, etc.), EEG is capable of recording brain activity at minuscule increments of time. Hence, EEG constitutes a favourable candidate for investigating the consumers’ brain activity. Moreover, recent advancements in the fields of neuroengineering, signal processing and machine learning have enabled neuroscientists and EEG practitioners to accurately decode users’ brain activity as registered by an EEG device. More specifically, researchers have managed to uncover neural signatures inextricably connected to cognitive aspects that are of particular interest in the context of marketing studies^5^. Motivated by the previous, EEG was the neuroimaging modality of choice incorporated in our experimental protocol.

However, despite the valuable insights that EEG may bring in investigating the consumers’ underlying neural processes, it cannot provide answers to more complex questions, like “which part of the advertising flyer captured the consumers’ attention?”^6^. Although attention is a cognitive process that could be potentially investigated by means of EEG, the identification of the exact object that draws a consumer’s attention is a task that cannot be supported by EEG-based metrics. In order to mine such information at a fine level of detail, EEG-scanners should be complemented by various physiological and behavioural monitoring tools^7^. By adopting such multimodal recording schemes, neuromarketing has actually advanced to a new era^6^. Aligned with the current tendency, our experimental protocol also employed eye-tracking (ET) and computer mouse data (position and clicks). Time-evolving observations from all these modalities were complemented with responses to questionnaires (containing demographic, profiling and product related questions) that represented the classical data-collection approach to marketing research.

Despite the plethora of EEG-related neuromarketing studies, the vast majority is based on inhouse datasets, with only two of them being publicly available^8,9^. The first one concerns product selection and contains only EEG data (14 electrodes), while the second one concerns advertisement appraisal solely from EEG sensors located mainly over the frontal brain region. On the contrary, we provide access to a multimodal neuromarketing dataset that revolves around the simulation of a realistic buying procedure from the catalogue of a real grocery store. Beyond the EEG signals, with 19 electrodes covering the whole 10–20 system, our dataset also includes eye-tracking and computer mouse data. Moreover, we provide responses to questionnaires that contain information with respect to each of the participants’ personality traits and demographics as well as individual product-related questions. The dataset that accompanies this paper contains both behavioural and physiological data that will enable researchers to investigate several marketing concepts (perception, decision making, product appraisal, etc.) from a neuroscientific perspective in a holistic manner. It should be noted that the purpose of this dataset is to promote exploratory research in the field of neuromarketing, hence, strict statistical significance tests should be employed in order to ensure generalizable and reliable findings.

## Methods

### Experimental Protocol

The experimental paradigm simulated the browsing experience of a digital advertising brochure, where the participants had to select the products they intended to buy. The paradigm was designed accordingly so as to monitor the subject’s brain and ocular activity throughout the browsing experience, with the original scope being the identification of distinct brain and ocular activity patterns with respect to selected (i.e. intended to buy) and non-selected products.

**Fig. 1:**
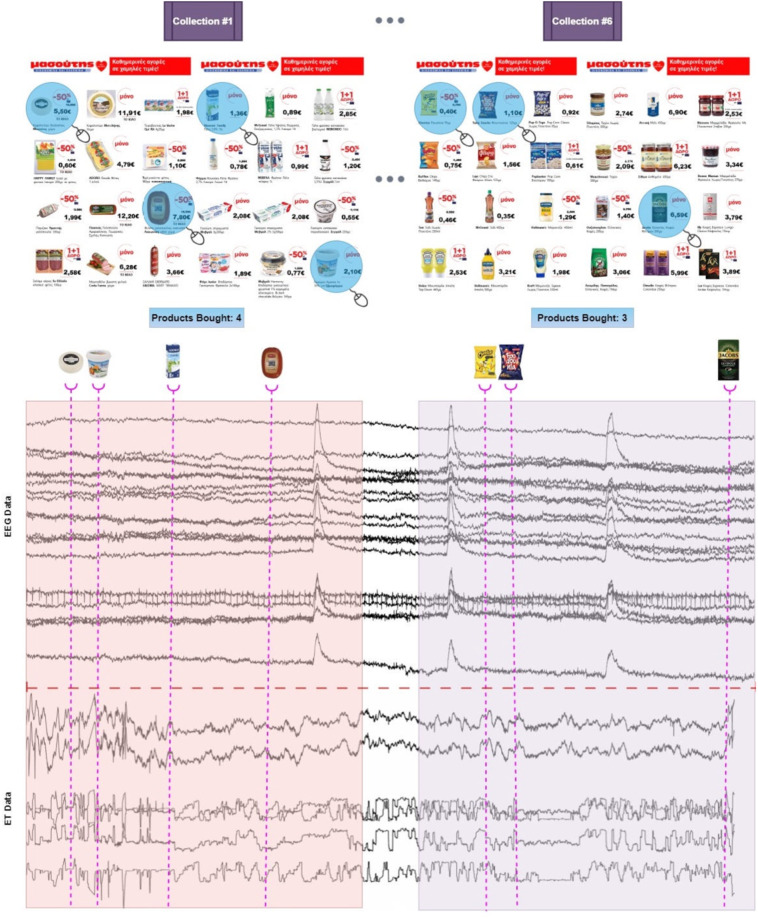
(**a**) The NeuMa-dataset experimental protocol/timeline. Six brochure pages including supermarket products were provided to the participants, that had to select products without any restriction. (**b**) The simultaneously recorded EEG activity and the corresponding mouse clicks embedded in the timeline of EEG traces.

During the experimental procedure, participants were seated in a comfortable armchair placed 50 cm from a 28 inch LCD monitor. Throughout the entire process, subjects could move their head freely. However, they were instructed to confine their movements (including head) as much as possible in order to minimize the artifacts in the EEG signals, but in a manner that does not cause any discomfort that may affect their overall experience. Prior to the product presentation, resting state EEG was recorded for two minutes. Once the recording of resting state was completed, participants could freely browse among the provided brochures by pressing the keyboard’s left and right arrow to move forwards and backwards respectively. Aiming to replicate the layout of a standard advertisement leaflet that will naturally lead to a more realistic experience for the participants, brochure pages were organized in order to contain products from the same product category (e.g. the first page included dairy products, the second frozen products, etc.). In total 144 supermarket products were illustrated encompassed into 6 brochure pages (each containing 24 products). Participants were instructed to identify and select the products (by left clicking on) they intended to buy in accordance with their regular buying habits, without having any global restrictions with respect to either the cost or the total number of products bought (i.e. the total number of selected products and consequently the total cost could significantly vary among participants). We should note that amongst the 144 viewed products, on average 18 were clicked by each participant leaving 126 unclicked. Figure 1 illustrates an exemplar case of the experimental timeline and the simultaneously recorded data and events, as depicted in the upper and lower part of the figure respectively. The products selected by the subject are highlighted in a light-blue colour in the upper panel, and the corresponding mouse clicks timestamps are embedded in the data recording as depicted in the lower panel. Finally, the total number of products selected in each brochure page is also incorporated in the figure.

Once the browsing/product selection experience was completed (ended by the participants on their own time), each participant was asked to fill in a questionnaire. The questionnaire included demographic questions (e.g. age, marital status, education, etc.), profiling questions (e.g. the big five personality traits, impulsive buying behaviour, etc.) and specific questions related to each of the selected products (i.e. reasons of selection, familiarity towards the selected product and whether the selected product constitutes a frequent purchase or not).

### Participant demographics

**Fig. 2:**
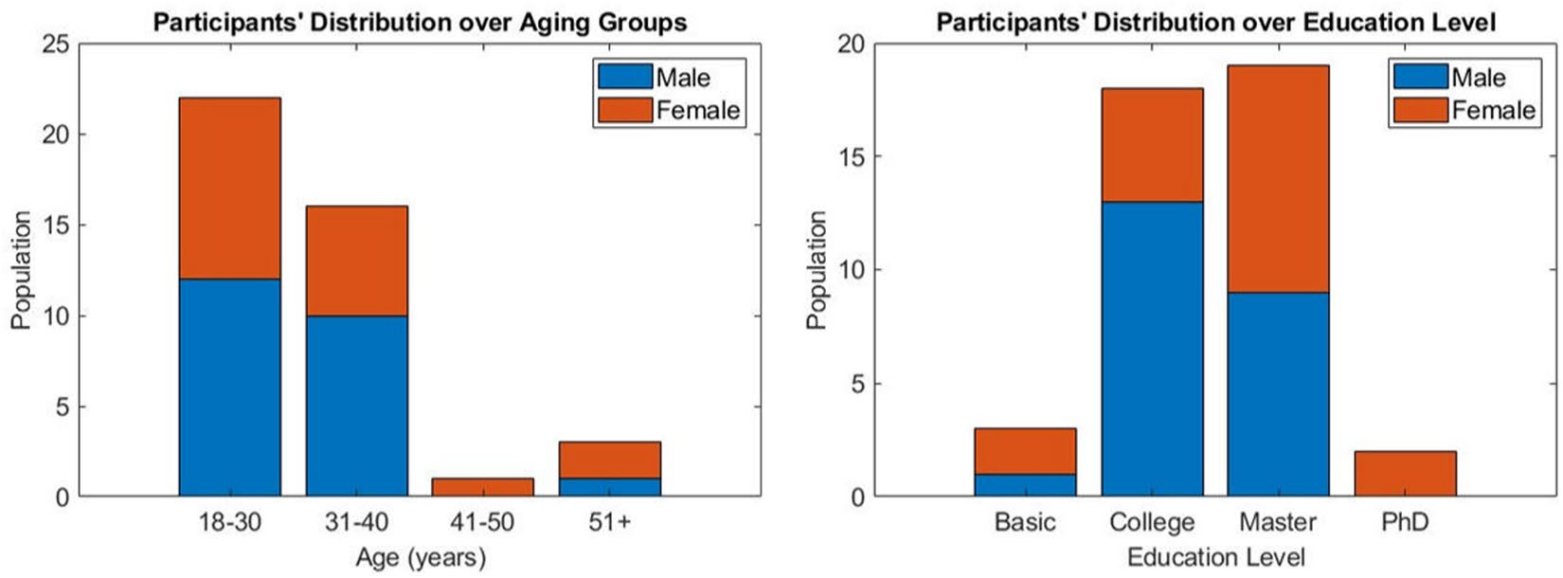
Participants’ distribution with respect to particular demographics (age and education level).

**Fig. 3:**
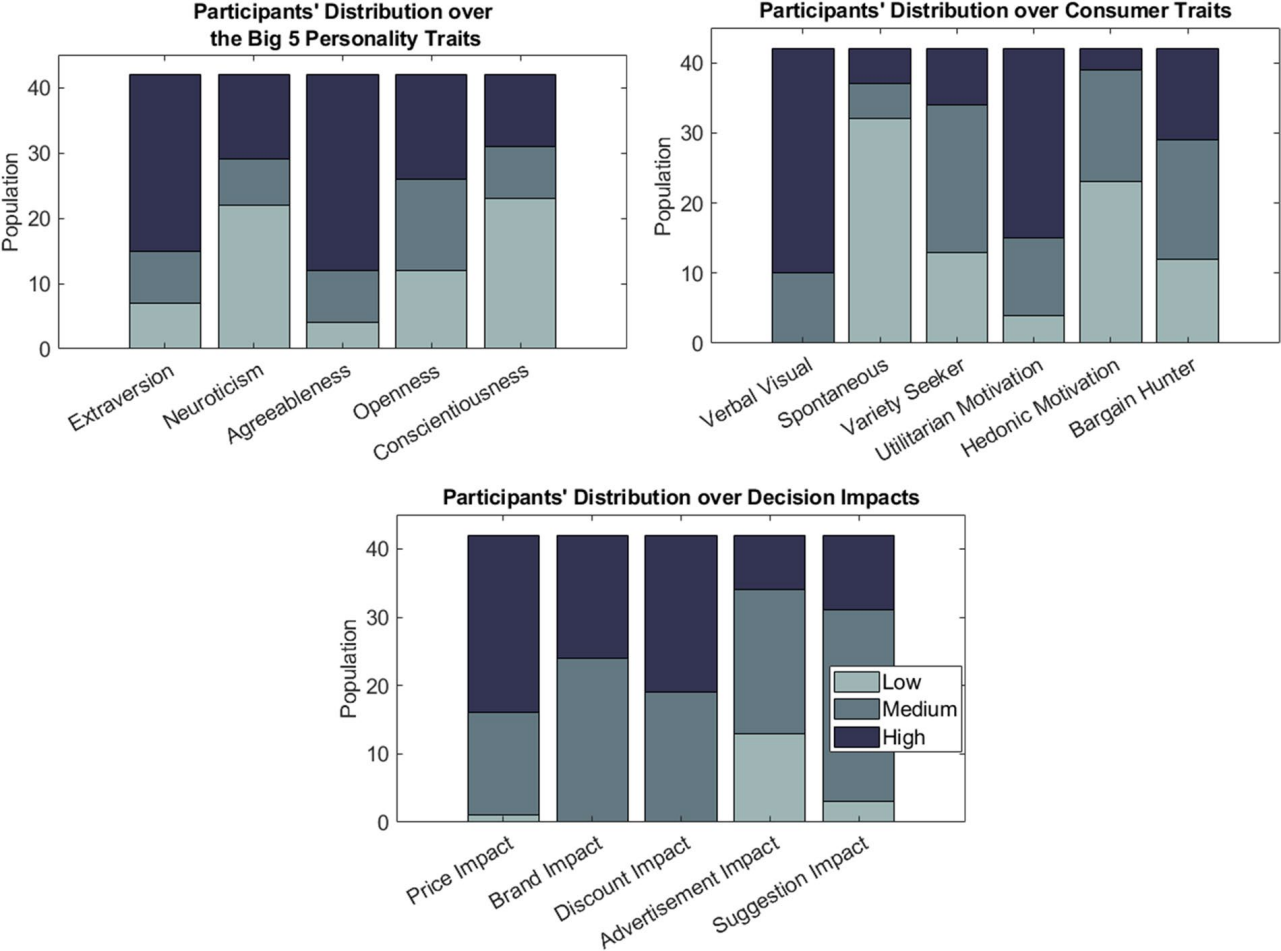
Participants’ distribution regarding profiling attributes. The low and high levels correspond to 25% and 75% of the measuring scale of the presented attributes accordingly.

A total of 45 individuals participated in this study, all native Greek speakers, originating from Greece. However, the dataset in its final form includes 42 subjects (23 males and 19 females, aged 31.5 ± 8.84), denoted as S01, S02, …, S42, as the data from three subjects were excluded due to high levels of artifactual contamination. Figure 2 provides the distribution of the participants’ demographics, with the left panel illustrating the age and the right the education level. As it can be seen, the distribution of participants’ age is skewed towards younger population. This fact is the aftereffect of limitation imposed by the experimental protocol, where the recruitment process was confined to participants exhibiting increased familiarity with computer interaction. Beyond that, this skewness aligns well with recent statistics which indicate that the demographics for online grocery consumers are concentrated over younger ages. Additionally, we note that amongst them, 34 are single, 7 are married and 1 is divorced, while 7 of the participants had at least one child. Figure 3 incorporates the distribution of the analysis of the responses to the profiling questions, including the Big 5 personality traits^10^, various consumer traits (e.g. utilitarian/hedonic motivation)^11–16^ and decision impacts. Prior to the recording, subjects were thoroughly informed about the experimental procedure and gave written informed consent that was approved by the Ethical Committee of the Centre for Research & Technology Hellas (CERTH), with Ref. No. ETH.COM-68.

### Problem formulation

The paradigm was designed to measure and study the subjects’ behavioural, ocular and brain responses during the decision making process. It naturally induced two different states (or conditions), hereafter referred to as “Buy” and “NoBuy” condition, the contrast of which is expected to reveal the essence of buying behaviour. The former condition includes the physiological responses when the participants were examining products they intended to buy. Whereas the latter condition incorporates the responses corresponding to when the participants were examining products they did not intend to buy. However, considering that the experimental scenario is of a dynamic nature and that detailed questionnaire responses are registered for each individual, the dataset can be employed to investigate various alternative scenarios, including but not limited to the following: (i) compare responses of competitive products (e.g. mustard of brand A vs mustard of brand B), (ii) compare the different consumer profiles generated via the questionnaire analysis (e.g. impulsive buyer, bargain hunter etc.) in terms of buying habits and brain/ocular activity patterns, (iii) examine what is the primal point of interest for the participants and whether they focus on the product image, textual description or price given their individual profiles, (iv) investigate the decision making processes of products characterised as “Bought” and the corresponding influential factors such as price, brand, and discount.

## Data Records

The dataset is provided into two forms, with the first including the raw experimental data (i.e. EEG data, ET data and mouse-related data) and the corresponding behavioural responses (i.e. questionnaires), hereafter referred as NeuMa Raw Dataset^17^, and the second its pre-processed version, referred as NeuMa Pre-processed Dataset^18^. Additionally, the raw version of the dataset is also provided in BIDS format^19^ that can be retrieved via the OpenNeuro repository^20^.

### Data recording and storage

#### NeuMa raw dataset

The recorded raw experimental data for each subject is stored in an Extensible Data Format (XDF) format, a general-purpose container format tailored for multi-channel biosignal time-series (e.g. EEG, ET, etc.) with extensive associated meta information. Each raw file is named after the subject ID (e.g. S01.xdf) and includes the following five data streams that were timely synchronised using LabRecorder (the recording program supported by Lab Streaming Layer, https://github.com/labstreaminglayer/App-LabRecorder): (i) the EEG data, (ii) the eye tracker data, (iii) the computer mouse clicks, (iv) the cursor positions on the screen controlled via the mouse, and (v) the markers. Each data stream is organised as a struct and besides the recorded data and timestamps also provides specific information about each stream (e.g. sampling frequency, device name, stream type, etc.). Importing XDF data in a programming framework (such as Matlab or Python) requires the use of the corresponding XDF modules (https://github.com/xdf-modules), while the type of each stream can be easily retrieved by the provided stream info, as illustrated in the *RawDataProcessing.mlx* file. A brief description for each data stream and the associated data is provided bellow:

- **EEG Data**: refers to the brain activity recorded in a continuous mode. The data structure consists of four fields. The first two, namely *info* and *segments*, provide information regarding the recorded data, with the most crucial information being stored under *info/nominal_srate* and *info/desc* providing information regarding the sampling frequency and the names of the recording electrodes (according to the 10–20 international system) respectively. The other two, namely *time_series* and *time_stamps*, encompass the recorded EEG activity ([number of electrodes × samples]) and the corresponding time stamps (unix timestamps).
- **Eye Tracker Data**: corresponds to the recorded gaze data, with the data structure being identical to the structure of the EEG data, except from the field *time_series*, where the provided information reflects the metrics for the left and right eye respectively. More specifically, the first three rows encompass data related to the left eye (i.e. 2D coordinates and pupil size) and the next three related to the right eye.
- **Mouse Clicks**: includes the sequence of clicks pressed, with the field *time_series* providing information regarding the type of click (i.e. left or right).
- **Mouse Positions**: includes the positions (i.e. 2D screen coordinates) for each mouse click included in the stream *Mouse Clicks*. Consequently the field *times_series* encompasses the 2D coordinates where each click took place.
- **Markers**: includes information regarding the alterations among brochure pages, with the field *time_series* providing the ID of the image collection (ranging between 1 and 6) and the field *time_stamps* the starting time of each presented brochure page. Finally, the values *fixation_cross* and *EOE (end of experiment)* encountered in the field *time_series*, refer to the initiation and completion of the experimental process respectively. Prior to the beginning of the browsing experience, there was a two-minutes long resting state recording period, during which the participants were looking at a fixation cross).

The registered behavioural responses for each subject are provided in a .xls file that follows the same naming convention as previously described for the .xdf files. Each .xls file includes the following information:

- **Demographics:** A series of demographic indicators namely, age, gender, education, marital status, children (boolean variable) and dominant hand.
- **Personality traits:** A series of clustered profiling related responses that upon analysis can provide information with respect to the following: (i) Big five personality traits^10^, (ii) Utilitarian/Hedonic shopping motivation^11^, (iii) Visual/Verbal information processing^12^, (iv) Impulse buying behaviour^13^, (v) Variety seeking behaviour^14^ and (vi) Bargain Hunting^16^.
- **Consumer Behaviour**: Information related to the participant’s buying behaviour, including: (i) Buying impact factors (i.e. in what extend the product selection is affected by (a) the product’s price, (b) the product’s brand, (c) the applied discount, (d) various product related promotional actions and (e) family/friend recommendation), (ii) Total number of weekly supermarket visits/Visit duration and (iii) The use or not of shopping list.
- **Product Info:** Information related to the products illustrated to the participants. In detail, for each product a broad description regarding the product is provided (e.g. Milk, Cereals, Biscuits, etc.) that is accessed via the file *Leaflet_Product_Descriptions.mat*. Additionally, for each selected product the responses regarding the following questions are provided:
  - Why was the product selected? - The possible responses regarding this question were: **A – Price**: The product was selected due to its price, **B – Brand**: The product was selected due to its brand, **C – Discount**: The product was selected because it was on a discount, **D – Type**: The selection was based on the product’s type (e.g. The participant was in need of milk and therefore selected one of the available milks), **E – Need**: The consumer was running out of the selected product and therefore was in need of it, **F – Like**: This was a product that the participant likes, **G – Regular**: This was a product that is regularly bought by the participant, **H – Combination**: This product was selected with the purpose of being combined with another selected product (e.g. cereals were selected, due to the previous selection of milk), **I – Alternative**: The product was selected as it was the closest alternative choice available (e.g. milk of brand A was selected, as milk of brand B was not available), **J – Other**: The product was selected for a reason that is not included in the previous options
  - Is this a familiar product? - The response to this question indicates if the product is frequently bought by the participant (**A - Yes**) or not (**B - No**).
  - Does this product constitute a frequent selection? - The response to this question indicates how familiar the participant is with the specific product. The responses range in a five-point scale (i.e. (**A - totally unfamiliar**) - (**E - completely familiar**)).

It is important to note here, that an additional .xls file that provides the encoding regarding the previously described information can be found under the name of *Questionnaire_Coding.xls*.

Additionally, the EEG electrode location coordinates and other electrode related information can be retrieved via the *chanlocs_dsi24.mat* file. Finally, the images of the 6 brochure pages used in the experimental process alongside with the bounding box coordinates of each product are provided as separate files. More specifically, the .tiff images are provided using the naming convention [*ImagePage_1.tiff, ImagePage_2.tiff, …, ImagePage_6. tiff]* under the folder *Brochure_Pages*, while coordinates are provided via.mat files in the form *[BoundingBoxPage_1.mat, BoundingBoxPage_2.mat, …, BoundingBoxPage_6.mat]* under the folder *BoundingBox_Coordinates*.

#### NeuMa pre-processed dataset

The pre-processed dataset besides the pre-processed timeseries for both the EEG and the ET datastreams, also provides the data segmentation per product, various product related information and the data extracted from the questionnaire analysis. Again the data is provided for each subject independently in a.mat file, under the same file naming convention described for the raw dataset. Additionally, the transition from the raw dataset to the pre-processed can also be achieved using the provided Matlab Live Script that can be accessed via Github (NeuMa Pre-Processed Dataset, https://github.com/NeuroMkt/NeuMa_Dataset_Processing) under the name *RawDataProcessing.mlx*. Finally, a second Matlab Live Script that provides general guidelines for handling and accessing the pre-processed data can also be found on our Github repository, under the name PreprocessedDataHandling.mlx. In the following a brief description regarding the data and information provided for each subject is provided:

- **The preprocessed EEG and ET data streams**. In the first stream, raw EEG signals were subjected to a bandpass filter (butterworth, 3rd order, zero-phased) within 0.5–45 Hz, followed by the removal of artifactual activity via the combined use of Artifact Subspace Reconstruction (ASR)^21^ and FORCe^22^. In the second (eye tracking), linear interpolation was applied to the gaze data in order to fill in missing data points (taking place during spontaneous eye blinking). Additionally, the sampling frequency (Fs) and the channel information (chans) for each data stream is provided. Finally, for each subject, the pre-processed data streams for EEG and ET are stored in variables *EEG_clean.data* and *ET_clean.data* respectively.
- **Information for each product**. The information is provided in the form of *PageNumber/ProductNumber* and includes the following info for each product:
  - Product Info: Incorporates the label regarding the “Buy” – “NoBuy”categorization (variable *bought*: {Buy - 1; NoBuy - 0}). Each product is also associated with three extra variables, namely *Reasons, Familiarity* and *FrequentBuy* answering the questions “Why was the product selected?”, “Is this a familiar product?” and “Does this product constitute a frequent selection?” respectively. In the case the product was not selected (i.e. *bought* = 0) all the aforementioned variables are set to zero. Finally, under the variable *Description*, the description for each product is provided (e.g. Milk, Cereals, Biscuits, etc.)
  - EEG segments: Provides the sample indices (in the form [sample start, sample_end]) of the continuous EEG recording (i.e. variable *EEG_clean.data*) that correspond to the time during which the participant spent looking at each product as defined by the smallest bounding box that contains the whole product image.
  - ET segments: Provides the sample indices (in the form [sample start, sample_end]) of the continuous ET recording (i.e. variable *ET_clean.data*) that correspond to the time during which the participant spent looking at each product as defined by the smallest bounding box that contains the whole product image. Additionally, the interested reader may also refer to our GitHub repository, where the tools for alternative segmentation strategies (as functions implemented in Matlab that can operate on the provided data streams) are provided. The aforementioned functions that facilitate the extraction of eye-related metrics (such as fixations and dwell time) can be combined with the stream conversion function (i.e. *converterET2EEG.m*) to examine the dataset from different viewpoints.

- **Personalised information collected via the questionnaires**. The information gathered for each subject are separated into two main categories:
  - Demographics - The demographic indicators for each subject (as described in the raw dataset).
  - Profile: The analysis of the questionnaire responses under the categories *Personality Traits* and *Consumer Behaviour*, resulted in the aggregation of the following general measures portraying each participant: (i) Big five personality traits, (ii) Utilitarian/Hedonic shopping motivation, (iii) Visual/Verbal information processing, (iv) Impulse buying behaviour, (v) Variety seeking behaviour, (vi) Bargain Hunting, (vii) Buying impact factors (including a score for price, brand, discount, advertisement and suggestion impact), (viii) Total number of weekly supermarket visits/Visit duration and (ix) Employment of a shopping list (boolean variable).

Finally, the brochure images and the bounding box coordinates are provided in the same way with the raw dataset.

**Fig. 4:**
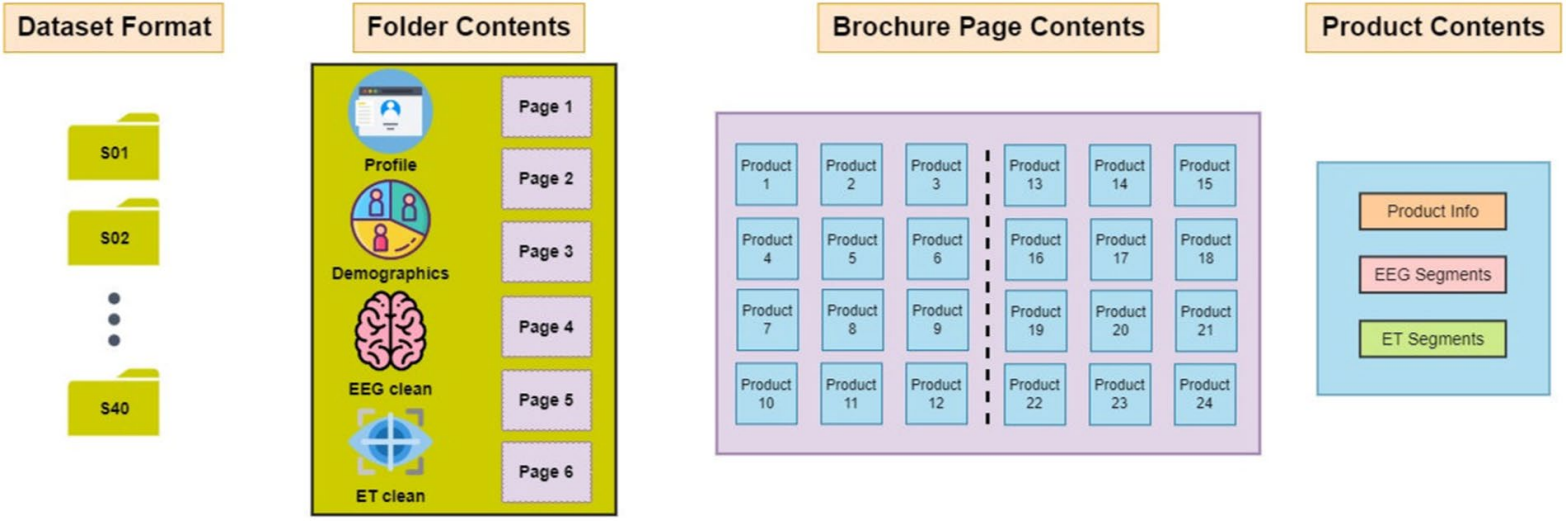
Illustration of the NeuMa Preprocessed Dataset format.

The format of the NeuMa Pre-processed Dataset, is graphically illustrated in Fig. 4, where the data and information provided for each subject, as described in this subsection, are depicted.

## Technical Validation

A total of 42 subjects that had normal or corrected-to-normal vision and none of them had taken any psycho-active or psycho-tropic substance, participated in the experimental process. Additionally, none of them reported a history of psychiatric disorders, neurological disease or drug use disorders.

**Fig. 5:**
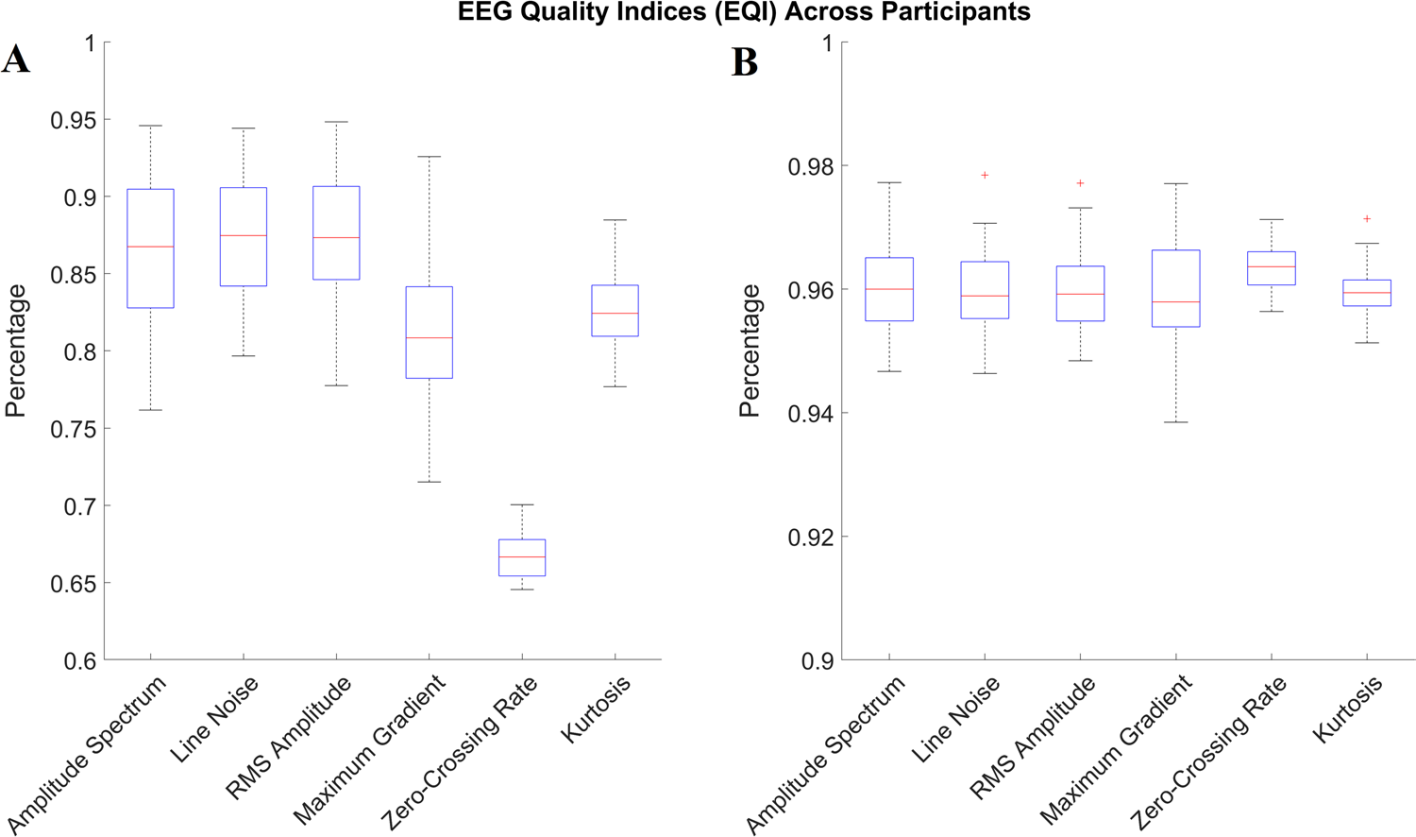
Percentage of EEG recording characterized by excellent (**a**) and good quality (**b**), based on the EEG Quality Indices.

**Fig. 6:**
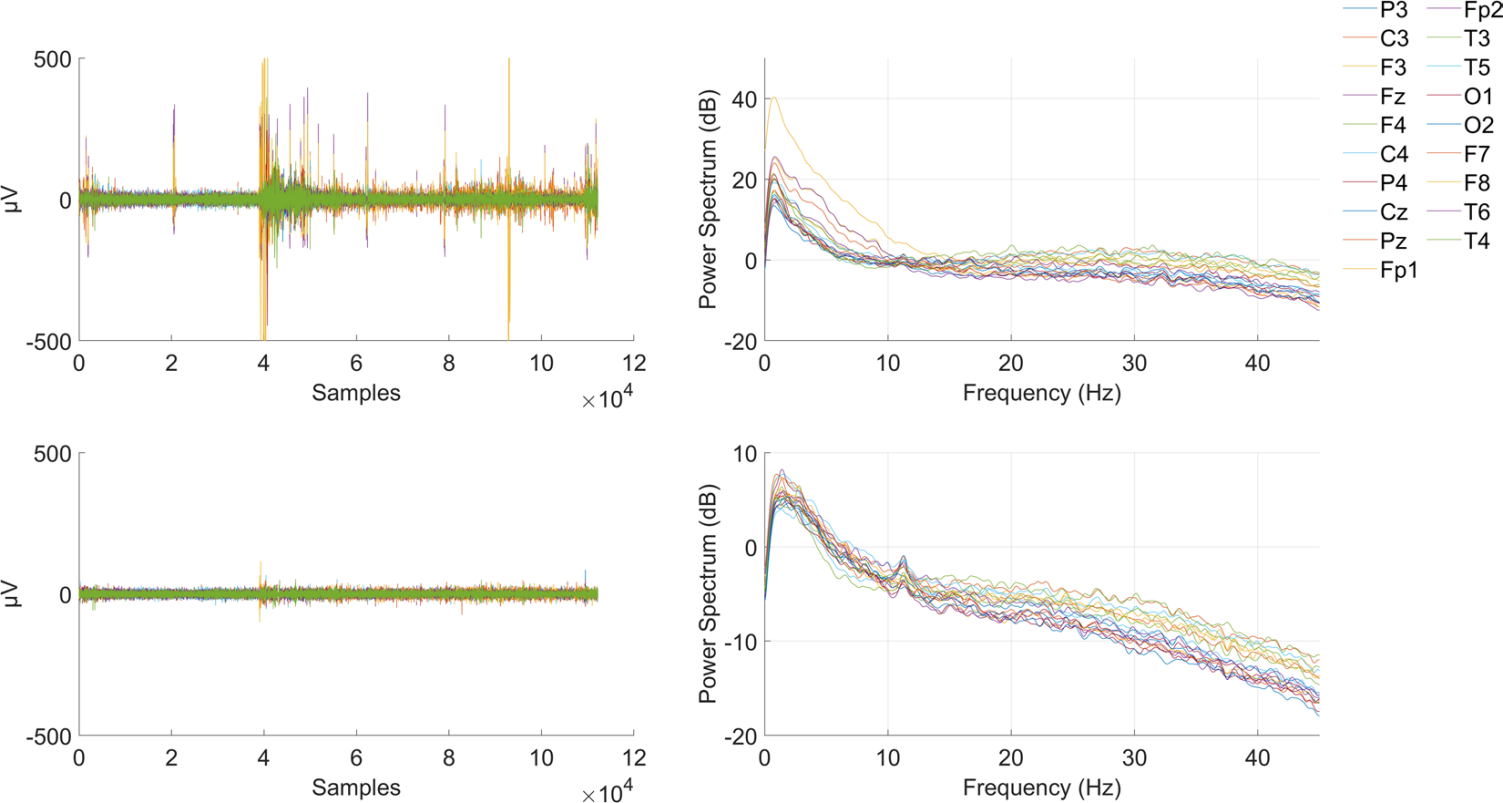
EEG recordings and the corresponding power spectrums regarding subject S14 for the raw (upper panel) and the pre-processed (lower panel) versions of the dataset.

The raw data was recorded using Wearable Sensing’s DSI 24 (https://wearablesensing.com/products/dsi-24/) and Tobii Pro Fusion (https://www.tobiipro.com/product-listing/fusion/) for the EEG and ET data respectively. The brain activity was recorded, with a sampling frequency of 300 Hz, via 21 dry sensors, namely Fp1, Fp2, Fz, F3, F4, F7, F8, Cz, C3, C4, T7/T3, T8/T4, Pz, P3, P4, P7/T5, P8/T6, O1, O2, A1 and A2, that were placed at locations corresponding to the 10–20 International System. The Sensors A1 and A2 were the reference electrodes $\left(\frac{A1+A2}{2}\right)$ and were placed on the mastoids. Prior to the experimental procedure, impedance for all electrodes was set below 10KΩ and EEG signals were inspected to avoid any irregularities. Additionally, an offline quality control was performed for the recorded data of each subject. More specifically, we employed the EEG Quality Indices (EQI) as proposed by Fickling *et al*.^23^, in order to assess the quality of the raw EEG signals. Hence the recorded EEG signals are segmented into 1-second-long non-overlapping windows and each window is being rated (i.e., excellent, good, poor, bad) according to its quality as defined by the EQI. As it can be seen in Fig. 5, approximately 80% of the total EEG recordings can be characterized as of excellent quality, whereas approximately 95% is at least of good quality. The remaining 5% corresponds to artifacts that were removed in the preprocessing stage, the steps of which are provided in Section *NeuMa Pre-processed Dataset*. Moreover, the recorded EEG data and their spectra were visually inspected upon the application of pre-processing steps (i.e. band pass filtering and artefact removal) and were found to be in line with the expected/typical encephalographic activity. An exemplar case for a randomly chosen subject (i.e. S14) is provided in Fig. 6, while the spectra for the entirety of the dataset can be accessed via the Figshare repository. The gaze data was recorded via Tobii’s screen based solution, with the eye movements being captured at a sampling frequency of 120 Hz. Prior to initiation of the experimental procedure, a 9-point calibration was performed for each subject, using the Tobii Pro SDK (https://www.tobiipro.com/product-listing/tobii-pro-sdk/), to formulate the individual’s eye model and gaze point. Finally, it should be noted that the selected products reported in the questionnaires were cross-checked with the ones registered via the corresponding mouse clicks.

## Usage Notes

The experimental datasets can be accessed and downloaded from the publicly accessible repository of Figshare, without mandatory registration, under the name NeuMa Raw Dataset and NeuMa Pre-processed Dataset. The data analysis can be performed either on Matlab or Python as implementations for loading .xdf files are available for both programming languages. However, we recommend the data analysis to be performed in Matlab, as several modules and functions have been already deployed by the authors of this dataset and can be accessed via GitHub (https://github.com/NeuroMkt/NeuMa_Dataset_Processing) Concluding, we strongly recommend the employment of both Live Scripts (*.mlx* files) as they demonstrate the practical usage of the dataset in a comprehensive manner.

In the following, we provide the key steps/instructions with respect to data loading, parsing and processing, as well as all the required steps that enable the transition from the raw dataset to its pre-processed version. The aforementioned steps have also been incorporated in the Matlab Live script named *RawDataProcessing.mlx*, that can be found in our Github repository:

- Data Loading: Loading an .xdf file either in Matlab or in Python requires the corresponding xdf module.
- Data Parsing: Once the loading process is completed, five separate datastreams registered as structs are available in the workspace. Hence, a data parsing process is required to identify the exact type of the stream, with the required information being stored in the field *info* for each struct.
- Preprocessing on EEG Data: The function *EEG_preprocessing.m*, incorporates the preprocessing steps, including but not limited to bandpass filtering and artefact rejection, applied on the continuous EEG recording.
- Preprocessing on ET Data: The linear interpolation performed to the gaze data is utilized by the function *interpNANs.m*.
- Segmentation: The EEG an ET signals are segmented on a product basis. Therefore, for each product, we provide the EEG and ET segments that correspond to the time intervals during which the participants’ gaze was located within the bounding box that encloses each product. These segments can be used in order to isolate the brain and ocular responses corresponding to particular products. The segmentation process includes two steps that are executed in sequence and are incorporated in the function *segmentationLeaflet.m*. Firstly, the data-stamps from the stream *Markers* alongside with the bounding boxes’ coordinates are exploited to define a brochure page and a product respectively. Then, the gaze coordinates are used to identify the segments (if available) during which the participant was observing each product.
- Product Labelling: Firstly, the mouse streams are exploited to identify the products that were selected by the participants. Then, the questionnaires were analysed in order to provide additional information (i.e. reasons of selection, familiarity with the product and buying frequency). It should be noted that the aforementioned additional information is provided only for the selected products. Finally, the category for each product is loaded from the corresponding file (*Leaflet_Product_Descriptions.mat*), so as to provide a rough description for each product. These are implemented in functions *segmentationLeaflet.m* and *parseExcelProfiles.m*.
- Stream Conversion: Provides the transition, in terms of samples, from the ET to the EEG datastream by converting the corresponding time aligned samples. The function that materialises the stream conversion can be found on the GitHub repository under the name *converterET2EEG.m*.

## Data Availability

The Matlab code developed for data loading, data parsing, segmentation and preprocessing required for the use of both datasets (provided via Figshare) alongside with two Matlab Live Scripts that demonstrate the data handling process for each dataset are provided in our Github repository (NeuMa Raw Dataset, https://github.com/NeuroMkt/NeuMa_Dataset_Processing/tree/main/NeuMa_Raw_Usage_Code; NeuMa Pre-processed Dataset, https://github.com/NeuroMkt/NeuMa_Dataset_Processing/tree/main/NeuMa_PreProcessed_Usage_Code).

## References

[1] Smidts, A. Kijken in het brein: Over de mogelijkheden van neuromarketing. (2002).

[2] Brammer M (2004). Brain scam?. Nature Neuroscience.

[3] Ozdemir M, Koc M (2012). Two methods of creative marketing research neuromarketing and in-depth interview. *Creative and Knowledge*. Society.

[4] Plassmann H, Venkatraman V, Huettel S, Yoon C (2015). Consumer neuroscience: applications, challenges, and possible solutions. Journal of marketing research.

[5] Hakim A, Levy DJ (2019). A gateway to consumers’ minds: Achievements, caveats, and prospects of electroencephalography‐based prediction in neuromarketing. Wiley Interdisciplinary Reviews: Cognitive Science.

[6] Kalaganis FP (2021). Unlocking the subconscious consumer bias: a survey on the past, present, and future of hybrid EEG schemes in neuromarketing. Frontiers in Neuroergonomics.

[7] Bercea, M. D. Anatomy of methodologies for measuring consumer behavior in neuromarketing research. In *Proceedings of the Lupcon Center for Business Research (LCBR) European Marketing Conference. Ebermannstadt, Germany* (2012, August).

[8] Yadava M, Kumar P, Saini R, Roy PP, Prosad Dogra D (2017). Analysis of EEG signals and its application to neuromarketing. Multimedia Tools and Applications.

[9] Hakim A (2021). Machines learn neuromarketing: Improving preference prediction from self-reports using multiple EEG measures and machine learning. International Journal of Research in Marketing.

[10] John, O. P. & Srivastava, S. The Big-Five trait taxonomy: History, measurement, and theoretical perspectives (1999).

[11] Babin BJ, Darden WR, Griffin M (1994). Work and/or fun: measuring hedonic and utilitarian shopping value. Journal of consumer research.

[12] Childers TL, Houston MJ, Heckler SE (1985). Measurement of individual differences in visual versus verbal information processing. Journal of Consumer Research.

[13] Rook DW, Fisher RJ (1995). Normative influences on impulsive buying behavior. Journal of consumer research.

[14] Rohm AJ, Swaminathan V (2004). A typology of online shoppers based on shopping motivations. Journal of business research.

[15] Cox AD, Cox D, Anderson RD (2005). Reassessing the pleasures of store shopping. Journal of Business research.

[16] Park YS, Lee SI, Choi I (2009). A study on the consumer’s service quality perception based on the types of life-style. Journal of Global Scholars of Marketing Science.

[17] Georgiadis K (2023). figshare.

[18] Georgiadis K (2023). figshare.

[19] Pernet CR (2019). EEG-BIDS, an extension to the brain imaging data structure for electroencephalography. Scientific data.

[20] Georgiadis K (2023). NeuMa OpenNeuro.

[21] Blum S, Jacobsen NS, Bleichner MG, Debener S (2019). A Riemannian modification of artifact subspace reconstruction for EEG artifact handling. Frontiers in human neuroscience.

[22] Daly I, Scherer R, Billinger M, Müller-Putz G (2014). FORCe: Fully online and automated artifact removal for brain-computer interfacing. IEEE transactions on neural systems and rehabilitation engineering.

[23] Fickling, S. D., Liu, C. C., D’Arcy, R. C., Hajra, S. G. & Song, X. Good data? The EEG quality index for automated assessment of signal quality. In *2019 IEEE 10th Annual Information Technology, Electronics and Mobile Communication Conference (IEMCON)* (pp. 0219–0229) (2019, October).

